# Development of a behavioural theory-based New Medicine Service toolkit for community pharmacists to promote medication adherence

**DOI:** 10.1007/s11096-025-01959-3

**Published:** 2025-06-28

**Authors:** Betul Okuyan, Pınar Ay, Mesut Sancar, Vildan Ozcan, Ozge Durak-Albayrak, Meltem Turker, Arman Uney, Corrine I. Voils

**Affiliations:** 1 Department of Clinical Pharmacy, Faculty of Pharmacy, Marmara University, Istanbul, Türkiye; 2 Department of Public Health, School of Medicine, Marmara University, Istanbul, Türkiye; 3 Turkish Pharmacists’ Association, Ankara, Türkiye; 4 Department of Internal Medicine, School of Medicine, University of Utah, Salt Lake City, UT USA; 5 William S. Middleton Memorial Veterans Hospital, Madison, WI USA

**Keywords:** Assessment of medication adherence, Behaviour change techniques, Cardiometabolic, Cardiovascular diseases, Diabetes mellitus, Nonadherence, adherence interventions, Theoretical domains framework

## Abstract

**Introduction:**

Adherence to newly prescribed cardiometabolic medications is low. It is crucial to develop personalized behavioural interventions to address patients’ specific medication adherence barriers.

**Aim:**

The aim of this study was to develop a behavioural theory-based New Medicine Service (NMS) toolkit for use by community pharmacists in Türkiye to identify and address reasons for nonadherence in patients newly started on medications for hypertension, diabetes, or dyslipidemia.

**Method:**

This multistage study used a literature search, expert panel, cognitive interviews, and survey with patients to identify reasons for nonadherence to include in the Turkish DOSE Nonadherence Scale. A short form of the toolkit was generated by asking the patients to select the most challenging reasons for nonadherence. To identify relevant pharmacist interventions, the theoretical domains framework (TDF-14) (v2) domains related to reasons for nonadherence were identified and matched with behavioural change techniques (BCTs). To assess the applicability of the proposed pharmacist interventions in daily practice, an online survey of community pharmacists was conducted using a modified Delphi study.

**Results:**

The final list of reasons for nonadherence consisted of 31 items, of which 14 identified as most challenging by patients were selected for inclusion in the short form of the NMS toolkit. For the full 31 reasons, ten domains of TDF-14 and 18 BCTs were selected. In the Delphi study (response rate: 83.3%), 68 of the 81 (84.0%) pharmacist interventions were found to be applicable, corresponding to 15 BCTs.

**Conclusion:**

The behavioural theory-based NMS toolkit was developed for use by community pharmacists to identify and address reasons for nonadherence in patients newly started on medications to manage hypertension, diabetes, or dyslipidemia. This toolkit will assist community pharmacists in developing personalized interventions to overcome nonadherence problems in patients who are newly starting medications. Future studies should be conducted to assess the impact of this new toolkit on patients' medication adherence levels and clinical outcomes.

**Supplementary Information:**

The online version contains supplementary material available at 10.1007/s11096-025-01959-3.

## Impact statements

- The NMS toolkit was developed for use by community pharmacists to screen for reasons for nonadherence and offer pharmacist interventions to address nonadherence issues.
- This toolkit will assist community pharmacists in developing personalized interventions to overcome nonadherence problems in patients newly starting cardiometabolic medications.

## Introduction

Medication adherence is *“the process by which patients take their medications as prescribed, composed of initiation, implementation and discontinuation”* according to the ABC (Ascertaining Barriers to Compliance) project [1]. One in three patients with chronic disease discontinue medication within 10 days of starting new medication [2]. A recent study showed that one in two patients stopped taking their medication 6 months after starting a new medication for a chronic condition (including cardiometabolic medications) [3]. Almost half of patients are nonadherent to cardiometabolic medications after one year of initiation [4].

Since 2011, the New Medicines Service (NMS) has been provided by pharmacists in the UK to patients receiving a first prescription for asthma, chronic obstructive pulmonary disease, hypertension, type 2 diabetes or anticoagulant/antiplatelet agents. This service consists of three parts: patient engagement, intervention, and follow-up. The intervention includes assessing medication adherence, identifying problems that contribute to nonadherence, and suggesting information and support for pharmacists to help overcome these problems [5]. Interview guides are available to standardize the intervention across pharmacists [6]. This service has been shown to improve the rate of adherence to newly initiated medicines [7].

The NMS is implemented in many European countries, such as in a transitional care setting for cardiovascular patients in the Netherlands [8], in community pharmacies in Norway for cardiovascular medications [9] and in Belgium for asthma patients [10].

Until recently, the number of adherence studies in Türkiye was low. The ABC taxonomy was recently translated into Turkish [11]. In one study, the rate of nonadherence to cardiometabolic medications was almost 30% [12]. In Türkiye, there are many postgraduate training programs about pharmaceutical services, including one on promoting medication adherence. For example, in a pilot study in 2019, the Turkish Pharmacists Association (TPA) developed a virtual continuing professional development program to promote medication adherence in older patients. Medication adherence increased in older patients who received theory-based pharmaceutical care service from community pharmacists participating in this virtual program [13]. However, all Turkish community pharmacists did not routinely provide a pharmaceutical care service to promote medication adherence in their daily practice. TPA has planned to implement the NMS in community pharmacies nationally.

Methods to promote medication adherence are diverse, complex, and have limited effectiveness [14]. Rather than a one-size-fits-all approach, there is need to develop a personalized, specific services to address the patient's specific medication adherence problems [15, 16]. Allemann et al. [16] suggested that matching potential medication adherence problems with appropriate interventions would result in effective strategies to overcome medication adherence barriers. Some tools consist of interventions to address the most commonly seen medication adherence problems in community pharmacies in the US [17, 18] and Spain [19]. The theoretical domains framework (TDF) [20, 21] and Behavioural Change Technique (BCT) [22, 23] frameworks have been used to develop structured interventions to improve medication adherence in older adults [24] and patients with bronchiectasis [25], stroke [26], and diabetes [27]. The TDF was created by synthesizing 33 theories to provide a single framework for identifying behavioral barriers and developing an intervention [20, 21]. The Behavior Change Technique Taxonomy (v1) consisted of 93 BCTs for use in behaviour change interventions [22, 23]. Recently, an intervention using TDF and BCTs has been developed to overcome the medication administration problems of family caregivers in Türkiye [28].

### Aim

The aim of this study was to develop a NMS Toolkit for use by community pharmacists in Türkiye to identify and address reasons for nonadherence in patients newly started on medications for hypertension, diabetes, or dyslipidemia. The study objectives were: (1) to adapt the Turkish version of the DOSE Nonadherence scale to include relevant reasons for nonadherence based on a literature search, expert panel (including a clinical pharmacist and physicians), and cognitive interviews and a survey with patients, (2) to determine possible pharmacist interventions by identifying relevant TDF-14 domains and selecting appropriate BCTs, and (3) to evaluate the applicability of pharmacist interventions in this toolkit by using a modified Delphi study among community pharmacists.

## Method

**Fig. 1:**
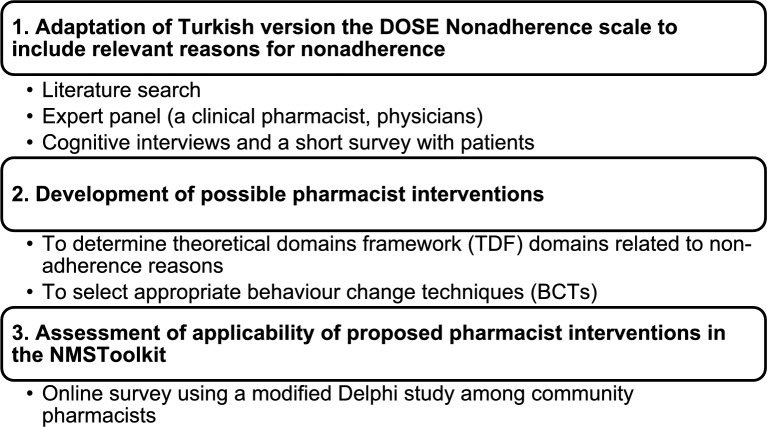
Study plan

The plan for this multiphase study is presented in Fig. 1.

### Adaptation of the Turkish version of the DOSE nonadherence scale to include relevant reasons for nonadherence

The DOSE-Nonadherence scale consists of two parts: extent of nonadherence and reasons for nonadherence. If patients report missed doses of medications on the first part, they complete the reasons of nonadherence part of the scale (18 items) [29–31]. Patients rate each reason for non-adherence according to its contribution to missing their dose (5-point Likert scale: from not at all to very much). While the extent of nonadherence items is universal and can be administered across populations and medication types, the reasons for nonadherence should be tailored for each patient population. Additional reasons can be based on existing literature or cognitive interviews with patients and clinicians. Initial lists of reasons for nonadherence have been published in the context of hypertension [29, 30], hepatitis C [31], diabetes [32], systemic lupus erythematosus [33], and cardiovascular medications [34].

The Turkish version of the DOSE-Nonadherence scale (including reasons for nonadherence for cardiometabolic conditions) was published in 2022 [12]. To identify additional reasons for this study, a literature search was conducted in April of 2023 using PubMed and Google Scholar. Search terms included (e.g. medication adherence, problem, barrier, chronic disease, noncommunicable disease, diabetes, hypertension, dyslipidemia). The final list of reasons for nonadherence consisted of 61 items.

An expert panel (n = 9; including a clinical pharmacist, three endocrinologists, a family physician, a cardiologist, two geriatricians, and one pulmonary disease specialist with expertise in the intensive care unit) was recruited through purposive sampling, aiming to have representation of various disciplines involved in the care of the patient population. They worked in six different tertiary university hospitals (male/female = 5/4). Most of them (n = 5) had 20 years or more of experience in their field. Between May–June 2023, the expert panel rated the necessity (content validity) of measuring each reason of nonadherence in patients with cardiometabolic disorders (hypertension, diabetes, dyslipidemia). Specifically, they scored the items as ‘’essential”, ‘’useful but not essential”, or ‘’not necessary.” For each item, the item content validity ratio (CVR) was calculated, and items were retained if the CVR was greater than 0.78, a threshold based on the number of experts according to Lawshe’s method [35]. The expert panel also suggested other important reasons for nonadherence not included in the list.

Cognitive interviews and a short survey with patients were conducted between October 2023 and January 2024 to evaluate the list finalized after expert panel and identify additional reasons from patient perspective. Using convenience sampling, patients were recruited from community pharmacies (located in different regions of Türkiye: Black Sea, Aegean, Mediterranean, Southeastern Anatolia). They were approached for enrollment any time they visited a community pharmacy within 1–8 weeks after receiving their new medication and provided written informed consent.

Patients were eligible if they were aged ≥ 18 years, had at least one cardiometabolic disorder (hypertension, diabetes, dyslipidemia), and were non-adherent to at least one newly prescribed medication (antihypertensive, hypoglycemic agents, statins) according to the extent of nonadherence items on the DOSE-Nonadherence scale, which was administered by trained community pharmacists. The extent of nonadherence items generated valid and reliable scores in previous study [30]. To be eligible, patients also had to be responsible for self-management of their medications. Patients completely dependent on assistance from others to take medications, as determined by the medication item on the Older Adults Resource Scale for Instrumental Activities of Daily Living [36], were excluded. Patients who had dementia and/or used antipsychotics or cholinesterase inhibitors or memantines were also excluded. Demographic data (age, gender, education, number of medications used, and indication for new medicine) were collected via survey. Lastly, trained community pharmacists conducted the cognitive interviews and a short survey including open-ended and closed ended [5-point Likert scale- 1 strongly disagree to 5 strongly agree] questions. The research team conducted online training sessions with the community pharmacists to conduct cognitive interviews using a structured interview guide and administer the survey.

The think-aloud technique with verbal probing was used to determine how an item was interpreted and a response formed [37]. Only reasons for nonadherence were assessed during this stage. During the interviews, other important reasons for nonadherence not originally included in the list were solicited. Following the interview, a short survey assessed their opinion on adding an open-ended question to identify additional barriers not listed. Participants were also asked to mark three reasons from the list that they found most challenging. Additional questions assessed patient perceptions of the use of this screening tool during pharmacy services. Their preferences about where and when this screening tool would be used (in a community pharmacy, by a follow-up call from a community pharmacist, or by using a mobile application) and their attitudes about completing this survey repeatedly over several weeks/months to help a community pharmacist understand how they are taking their medications over time were also explored.

### Development of pharmacist interventions

The steps proposed by French et al. [38] were followed to develop pharmacist interventions. Firstly, the problem was defined as non-adherent to newly prescribed medications in patients with cardiometabolic disease (including hypertension, diabetes, dyslipidemia). Initiation, implementation and persistence of newly prescribed medication (according to ABC taxonomy) were selected as the target behaviors. In the second step, each reason for nonadherence identified by literature review, expert panel and cognitive interviews with patients was matched by BO and CIV with at least one relevant TDF-14 domains [21]. Then, in third step, BO and CIV selected relevant BCTs [22, 23] according to the definition of BCTs and relevant TDF domain using the Theory and Techniques Tool (TTT) [39]. Pharmacist interventions were generated based on their content expertise and examples in previous studies [24–28, 40]. The entire research team then assessed the proposed pharmacist interventions, relevant TDF domains, and BCTs by assessing the APEASE (Affordability, Practicability, Effectiveness, Acceptability, Side effects/safety, and Equity) criteria [41] for each reason.

### Assessment of applicability of proposed pharmacist interventions in the NMS toolkit

Based on previous studies [42, 43], a two-round, online modified Delphi survey was conducted between November and December 2024 with community pharmacists (n = 30). The online survey was developed using the JotForm platform after pilot testing. Purposive sampling was used to ensure community pharmacists had Master of Science degree in clinical pharmacy and/or experience in pharmaceutical services to promote medication adherence in older adults [9] and to represent all geographic regions of Türkiye from network of the research team.

Electronic informed consent was obtained. Demographic data (gender, years of experience in community pharmacy, regions of Türkiye, attending to the postgraduate course/programs, having Master of Science/PhD, and availability of separate patient counselling room in the community pharmacy setting) were collected via online survey. The pharmacists were provided with a list of reasons for nonadherence, along with the relevant TDF-14 domain, selected BCTs, and pharmacist interventions that would address each reason. A 5-point Likert scale was used to assess applicability of each pharmacist intervention (1 = not at all applicable- 5- very applicable) considering practical aspects of the pharmacy workflow, workload, and patient needs. The pharmacists could provide additional comments via an open-ended question. The items that did not receive consensus agreement in the first round were modified by BO and MS according to the comments. Then, in the second round, the revised items were presented with the scores obtained from the first round. With a 5-point Likert scale (disagree strongly to agree strongly), agreement for each pharmacist intervention was defined as ≥ 70% of panelists scoring 4–5 and < 15% scoring 1–2 [42, 44].

### Ethics approval

Ethical approval was granted from Marmara University Medical School Clinical Trials Ethical Committee (no: 09.2022.945; date: 22.07.2022). Written informed consent was obtained from the participants.

## Results

### Adaptation of Turkish version of the DOSE nonadherence scale to include relevant reasons for nonadherence

In the content validity assessment, the expert panel suggested retaining 23 of the 61 reasons for nonadherence. They also recommended the addition of two additional reasons not included on the list (not remember whether they took their medicine and not get their medicine at the pharmacy).

Cognitive interviews and a short survey were conducted among 18 patients who were mostly female [n = 14] with education level less than 12 years [n = 13] (Table 1). Eight trained pharmacists from four different regions in Türkiye conducted the interviews and administered a short survey.

**Table 1:** Characteristics of the participants

Cognitive interviews and a short survey with patients (n = 18)	n (%)	Online survey using a modified Delphi study among community pharmacists (n = 25)	n (%)
Female	14 (77.8)	Female	16 (64.0)
Older adults (= > 65 years)	7 (38.9)	Years of experience in community pharmacy	
Indication for new medicine		< 10 years	4 (16.0)
Hypertension	8 (44.4)	11–20 years	9 (36.0)
Type 2 diabetes	7 (38.9)	> 20 years	12 (48.0)
Dyslipidaemia	3 (16.7)	Regions of Türkiye	
Education level Low (< 12 years*)	13 (72.2)	Marmara	6 (24.0)
Polypharmacy** (yes)	10 (55.6)	Aegean	4 (16.0)
Chronically medication use before new medicine prescription (yes)	10 (55.6)	Eastern Anatolia	4 (16.0)
		Black Sea	3 (12.0)
		Mediterranean	3 (12.0)
		Southeastern Anatolia	3 (12.0)
		Central Anatolia	2 (8.0)
		Attending to the postgraduate course/programs	25 (100.0)
		Having Master of Science/PhD	17 (68.0)
		Availability of separate patient counselling room in the community pharmacy setting	
		Yes	4 (16.0)
		Partially***	16 (64.0)
		No	5 (20.0)

*Duration of the compulsory education in Türkiye was 12

**Utilization 5 or more medications

***There was special place where they provide patient counselling inside the community pharmacy, but not a room

In the cognitive interviews, patients reported feeling uncomfortable about the reason related to the medication affecting their sex life. This reason was removed from the list. Seven new items were added based on patients’ suggestions, resulting in a list of 31 items for evaluation in patient cognitive interviews.

In a survey, 14 items (part 1- short form) were rated by patients as most challenging. Most participants (n = 8; 44.4%) stated they chose these items because of their higher incidence rate. Most (n = 12; 66.7%) agreed to add an open-ended question to explore any additional barriers. Most (n = 13; 72.2%) indicated that they would be willing to answer these items during their meetings with their pharmacist in a community pharmacy. Most (n = 13; 72.2%) also agreed to complete this screening tool repeatedly over several weeks/months to help a community pharmacist understand how they are taking their medications over time were explored. Some (n = 4; 22.2%) indicated that they could not use a mobile device to complete the measure.

### Development of pharmacist interventions

A total of 31 reasons for nonadherence (Tables 2 and Supplement File 1) were related to ten domains of the TDF-14 as follows: beliefs about consequences (n = 7), environmental context and resources (n = 6), social influences (n = 6), knowledge (n = 3), emotion (n = 3), reinforcement (n = 3), memory, attention, and decision processes (n = 2), skills (n = 2), beliefs about capabilities (n = 1), and goals (n = 1). Some reasons were related to both emotions and beliefs about consequences (n = 3). Eighteen 18 BCTs and 81 interventions were identified by two authors (BO and CIV) to address the reasons for nonadherence. The research team assessed each, and an accepted BCTs were retained. The selected BCTs were: 1.2. Problem solving, 1.4 Action Planning, 1.5. Review behaviour goal(s), 12.1. Restructuring the physical environment, 12.5. Adding objects to the environment, 2.3. Self-monitoring of behaviour, 2.4. Monitoring of outcome(s) of behaviour without feedback, 2.6 Biofeedback, 2.7. Feedback on outcome(s) of behaviour, 3.2. Social support (practical), 4.1. Instruction on how to perform behaviour, 5.1. Information about health consequences, 7.1. Prompts/cues, 7.7. Exposure, 8.1. Behavioural practice/rehearsal, 8.3. Habit formation, 9.1. Credible source, and 9.2. Pros and cons.

**Table 2:** Final version of New Medicine Service toolkit

Items I missed my dose because…*	TDF Domain	BCTs	Interventions
1. I did not know why I must take this medicine^#^	Knowledge	5.1. Information about health consequences	NMS#1.1. Provide information regarding the health consequences of (taking/not taking) medications based on the Health Belief Model by using reliable references and written materials or a QR code with relevant material
2. I did not know what the side effects or potential harm of this medicine were^#^	Knowledge	5.1. Information about health consequences	NMS#2.1. Provide information regarding potential side effects based on the Health Belief Model by using reliable references and written materials. If there is no time for counselling, provide the patient with written materials or a QR code of relevant material
3. I did not understand the physician’s/pharmacist’s instructions^#^	Knowledge	4.1. Instruction on how to perform behaviour	NMS#3.1. Provide information by using the teach-back method using written materials and a short video
		8.1. Behavioural practice/rehearsal	NMS#3.2. Prompt the patient to practice using an insulin pen in the community pharmacy
		1.4 Action Planning	NMS#3.3. Discuss medication scheduling with the patient according to their daily routine by providing a pill card and/or pill box
		12.5. Adding objects to the environment	NMS#3.4. Provide a pill card and/or pill box. NOTE: The patient's self-efficacy to use the pill box should be considered. Also, the pharmacist should assess the appropriateness of the pill box for the medications being used
4. I could not meet the food requirements (including empty stomach, taking with meals, no taking with alcohol)^#^	Skills	1.4. Action Planning	NMS#4.1. Discuss medication scheduling with the patient according to their daily routine (if the patient has no plan)
		12.5. Adding objects to the environment	NMS#4.2. Provide a medication schedule to the patient (including a pill card) and suggest using a pill box (if the patient has no plan). NOTE: The patient's self-efficacy to use the pill box should be considered. Also, the pharmacist should assess the appropriateness of the pill box for the medications being used
		8.3. Habit formation (If the patient has schedule/plan)	NMS#4.3. Advise the patient to integrate the behaviour with existing habits, if possible (if the patient has plan)
		4.1. Instruction on how to perform behaviour	NMS#4.4. Provide patient education and counselling by using the teach-back method. Discuss alcohol intake (including the amounts)
		7.1. Prompts/cues (If the patient has schedule/plan)	NMS#4.5. Advise the patient to set an alarm and place a sticker or magnet to remind them to take their medication
		12.1. Restructuring the physical environmental (If the patient has schedule/plan)	NMS#4.6. Advise the patient about where to put the pill box to promote medication taking, such as on the kitchen table or next to the bed
5. My treatment was so complicated, because I was on so many medications with different instructions^#^	Beliefs about capabilities	1.2. Problem solving	NMS#5.1. Provide a Brown Bag check-up by using the teach-back method Contact the physician to simplify the regimen
		1.4. Action Planning	NMS#5.2. If required, provide a medication schedule to the patient (including a pill card) and suggest using a pill box
		12.5. Adding objects to the environment	NMS#5.3. Provide a pill card and/or pill box. NOTE: The patient's self-efficacy to use the pill box should be considered. Also, the pharmacist should assess the appropriateness of the pill box for the medications being used
6. The medication caused side effects^#^	Reinforcement	1.2. Problem solving	NMS#6.1. Contact the patient’s physician to modify the treatment or refer them to their physician
		5.1. Information about health consequences	NMS#6.2. Provide information regarding management of potential side effects by using reliable references giving written materials or a QR code for relevant material
7. I did not remember to take my medicine^#^	Memory, attention, and decision processes	2.3. Self-monitoring of behaviour	NMS#7.1. Advise the patient to use a diary or checklist
		8.3. Habit formation	NMS#7.2. Advise the patient to integrate the behaviour with existing habits, if possible
		12.1. Restructuring the physical environmental	NMS#7.3. Advise the patient about where to put the pill box to promote medication taking, such as on the kitchen table or next to the bed
		7.1. Prompts/cues	NMS#7.4. Advise the patient to set an alarm and place a sticker or magnet to remind them to take their medication
		12.5. Adding objects to the environment	NMS#7.5. Advise the patient to use a pill box. NOTE: The patient's self-efficacy to use the pill box should be considered. Also, the pharmacist should assess the appropriateness of the pill box for the medications being used
		3.2. Social support (practical)	NMS#7.6. Advise asking a friend/family member to help the patient remember to take their medication. NOTE: If there is no time for counselling, provide the patient with written materials or a QR code with relevant material
8. I could not fill the prescription on time^#^	Environmental context and resources	3.2. Social support (practical)	NMS#8.1. Advise asking a friend/family member to help the patient take their medication. NOTE: If there is no time for counselling, provide the patient with written materials or a QR code of relevant material
		7.1. Prompts/cues	NMS#8.2. Advise the patient to set an alarm and place a sticker or magnet to remind them to take their medication
9. I could not get my medicine at pharmacy^#^	Environmental context and resources	1.2. Problem solving	NMS#9.1. Identify the reasons (such as medication shortage). Advise the patient to use generic medications and/or contact their physician
10. I could not remember whether I took my medicine or not^#^	Memory, attention, and decision processes	2.3. Self-monitoring of behaviour	NMS#10.1. Advise the patient to use a diary or checklist
		12.5. Adding objects to the environment	NMS#10.2. Advise the patient to use a pill box. NOTE: The patient's self-efficacy to use the pill box should be considered. Also, the pharmacist should assess the appropriateness of the pill box for the medications being used
11. The physician did not spend enough time with me and did not explain my treatment^#^	Social influences	5.1. Information about health consequences	NMS#11.1. Provide information regarding the health consequences of (taking/not taking) medications based on the Health Belief Model by using reliable references and written materials
12. I could not get answers to my questions about the medication^#^	Social influences	1.2. Problem solving	NMS#12.1. Ask the patient if they have questions and answer the questions. If required, refer them to their physician
13. I went to another physician, and this physician said I didn't need to use this medicine^#^	Social influences	2.4. Monitoring of outcome(s) of behaviour without feedback	NMS#13.1. Advise the patient to record their blood pressure/glucose daily or to have their lipid profile checked one month later
14. I am worried about side effects^#^	Emotion Belief of consequences	5.1. Information about health consequences	NMS#14.1. Provide the patient with counselling regarding potential side effects and their management using reliable references
15. I did not receive any practical training on how to use special medications (such as insulin pens)	Skills	4.1. Instruction on how to perform behaviour	NMS#15.1. Provide information by using the teach-back method using written materials and a short video
		8.1. Behavioural practice/rehearsal	NMS#15.2. Prompt the patients to practice using an insulin pen in the community pharmacy
16. I do not like needles	Emotion Belief of consequences	8.1. Behavioural practice/rehearsal	NMS#16.1. Check whether the patient took the medication correctly
		1.2. Problem solving	NMS#16.2. Contact/refer the patient to the physician to assess the medication treatment plan. (The physician would assess the use of oral medications instead of SC medication.)
		3.2. Social support (practical)	NMS#16.3. Advise asking a friend/family member to help the patient administer SC medications. NOTE: If there is no time for counselling, provide the patient with written materials or a QR code with relevant material
17. I hate to take the medicine	Emotion Belief of consequences	3.2. Social support (practical)	NMS#17.1. Advise asking a friend/family member to help the patient with subcutaneous administration of the medication. NOTE: If there is no time for counselling, provide the patient with written materials or a QR code with relevant material
18. I was afraid the medication would interact with other medication I take	Belief of consequences	5.1. Information about health consequences	NMS#18.1. Provide information regarding drug interactions and their management using reliable references
19. I thought treatment was over	Belief of consequences	1.5. Review behaviour goal(s)	NMS#19.1. Discuss treatment goals using national and international guidelines
		5.1. Information about health consequences	NMS#19.2. Discuss goals of medication taking. NOTE: If there is no time for counselling, provide the patient with written materials or a QR code with relevant material
20. The medication was not working	Belief of consequences	2.6 Biofeedback 2.3. Self-monitoring of behaviour 2.7. Feedback on outcome(s) of behaviour	NMS#20.1. Advise the patient to self-monitor clinical outcomes by using a glucometer and/or blood pressure monitor and recording the values Advise the patient to record daily medication taking using a diary Schedule a meeting to discuss the patient’s blood pressure and glucose values according to their weekly record Inform the patient about the decrease in their blood pressure/glucose when they take the medication and the increase when they do not NOTE: This intervention may be recommended for patients with an intentional (deliberate) non-adherence problem and/or patients with high health literacy
		5.1. Information about health consequences	NMS#20.2. Provide information about impact of the medications using reliable references
21. I did not think I needed the medicine	Belief of consequences	2.6 Biofeedback 2.3. Self-monitoring of behaviour 2.7. Feedback on outcome(s) of behaviour	NMS#21.1. Advise the patient to self-monitor clinical outcomes by using a glucometer and/or blood pressure monitor and recording the measures. Advise the patient to record daily medication taking using a diary. Schedule a meeting to discuss the patient’s blood pressure and glucose values according to their weekly record Inform the patient about the decrease in their blood pressure/glucose when they take the medication and the increase when they do not NOTE: This intervention may be recommended for patients with an intentional (deliberate) non-adherence problem and/or patients with high health literacy
		5.1. Information about health consequences	NMS#21.2. Provide information regarding the health consequences of (taking/not taking) medications based on the Health Belief Model
22. The injection causes pain	Reinforcement	5.1. Information about health consequences	NMS#22.1. Provide information about why the injection causes pain and potential side effects
		8.1. Behavioural practice/rehearsal	NMS#22.2. Check whether the patient applied correctly using the teach-back method
		1.2. Problem solving	NMS#22.3. Contact/refer the patient to the physician to assess the medication treatment plan. (The physician would assess the use of oral medications instead of SC medication)
23. The medicine was making my condition worse	Reinforcement	5.1. Information about health consequences	NMS#23.1. Provide information regarding the health consequences of (taking/not taking) medications based on the Health Belief Model and using reliable sources
		2.6 Biofeedback	NMS#23.2. Advise the patient to self-monitor clinical outcomes by using a glucometer and/or blood pressure monitor and recording the values
		2.3. Self-monitoring of behaviour	NMS#23.3. Advise the patient to record daily medication taking using a diary
		2.7. Feedback on outcome(s) of behaviour	NMS#23.4. Schedule a meeting to discuss the patient’s blood pressure and glucose values according to their weekly record. Inform the patient the decrease in their blood pressure/glucose when they take the medication and the increase when they do not
24. I was busy	Goals	1.4. Action Planning	NMS#24.1. Discuss medication scheduling with the patient according to their daily routine
		12.5. Adding objects to the environment	NMS#24.2. Provide a pill card and/or pill box. NOTE: The patient's self-efficacy to use the pill box should be considered. Also, the pharmacist should assess the appropriateness of the pill box for the medications being used
		8.3. Habit formation	NMS#24.3. Advise the patient to integrate the behaviour with existing habits, if possible
		12.1. Restructuring the physical environmental	NMS#24.4. Advise the patient about where to put the pill box to promote medication taking, such as on the kitchen table or next to the bed
		7.1. Prompts/cues	NMS#25.5. Advise the patient to set an alarm and place a sticker or magnet to remind them to take their medication
		3.2. Social support (practical)	NMS#25.6. Advise asking a friend/family member to help the patient remember to take their medication. NOTE: If there is no time for counselling, provide the patient with written materials or a QR code with relevant material
25. The medicine was too expensive	Environmental context and resources	1.2. Problem solving	NMS#25.1. Identify the reasons and solutions. Advise the patient to use generic medications and/or contact their physician
26. I did not have my medicines with me	Environmental context and resources	3.2. Social support (practical)	NMS#26.1. Advise asking a friend/family member to help the patient take their medication. NOTE: If there is no time for counselling, provide the patient with written materials or a QR code with relevant material
		7.1. Prompts/cues	NMS#26.2. Advise the patient to set an alarm and place a sticker or magnet to remind them to take their medication
		12.1. Restructuring the physical environment	NMS#26.3. Advise the patient to put the medications in the a bag/pill box or to keep spare medications
27. Suitable conditions for storing my medicine were not available (such as refrigerator, humidity)	Environmental context and resources	12.1. Restructuring the physical environment	NMS#27.1. Advise the patient to put the medications in the a bag/pill box or to keep spare medications
28. I did not have the supplies needed to take the medicine (e.g., syringes/needles)	Environmental context and resources	7.1. Prompts/cues	NMS#28.1. Advise the patient to set an alarm and place a sticker or magnet to remind them to take their medication
		12.1. Restructuring the physical environment	NMS#28.2. Advise the patient to put the medications in a bag/pill box or to keep spare supplies
29. There was no one who could help me take and use medications	Social influences	3.2. Social support (practical)	NMS#29.1. Provide pharmacist-led practical help Advise family/friends supporting the patient by giving them written materials or a QR code with relevant material
30. My family or friends suggested me not take the medicine	Social influences	5.1. Information about health consequences	NMS#30.1. Provide information regarding the health consequences of (taking/not taking) medications based on the Health Belief Model by using reliable references and written materials or a QR code with relevant material
31. I hesitated to ask my questions to the physician about the medicines	Social influences	8.1. Behavioural practice/rehearsal	NMS#31.1. Discuss how to best prepare and behave during healthcare visits. Ask the patient if they have questions and answer the questions. List the questions the patient could ask at their next physician appointment

NMS, New Medicine Service; SC, subcutaneous

*All items are translated from Turkish form

^#^Items included in short form

### Assessment of applicability of proposed pharmacist interventions in the NMS toolkit

Twenty-five pharmacists participated in the modified Delphi study (response rate 83.3%). Pharmacist characteristics are presented in Table 1. They assessed 81 pharmacist interventions for 31 reasons for nonadherence assessed by 25 community pharmacists.

Of the 81 proposed interventions, 46 (57%) received consensus agreement in Round 1. The community pharmacists' reservations were about discussing their support from family/friends with the patients, administering subcutaneous medications in the community pharmacy, having limited time to watch videos in the community pharmacy, perceived difficulties in creating the list with patients with low literacy, and the patients’ self-efficacy in using the pillbox. Some amendments were made for Round 2 (Supplement File 1), including a general note regarding all materials and resources about pharmacist intervention would be provided by TPA. In Round 2, the community pharmacists evaluated 35 pharmacist interventions that did not reach consensus in Round 1 (as < 70% of panelists scoring 4–5 and >  = 15% scoring 1–2). Of those, 22 (27%) proposed interventions reached consensus in round 2. Thus, following two rounds of testing, 68 pharmacist interventions (84.0%) with matching 15 BCTs were rated as applicable for implementation by community pharmacists (Table 2).

## Discussion

The NMS Toolkit was developed for use by community pharmacists to identify and address reasons for nonadherence in patients newly started on medications to manage hypertension, diabetes, or dyslipidemia. In the final version of the NMS toolkit, 68 pharmacist interventions, corresponding to 15 BCTs, were identified as applicable for 31 reasons for nonadherence related to ten TDF-14 domains.

There are many self-report tools to assess the extent and/or reasons for nonadherence [45], and some tools consist of interventions to address the most common medication adherence problems [17–19]. Recently, the Medication Adherence Barriers Questionnaire (IMAB-Q) was developed based on the TDF-12 (v1) to identify barriers related to medication adherence. The authors conducted a scoping review and focus groups among patients taking medications for the prevention of cardiovascular disease [46]. They stated that, by using the IMAB-Q, the appropriate BCTs could be selected to overcome the medication adherence barriers classified based on the TDF. Another research team generated the Test of Adherence to Inhalers (TAI) toolkit, which consists of evidence-based interventions to promote medication adherence that matched with the medication adherence barriers items in the Test of Adherence to Inhalers tool [47, 48]. The interventions in the TAI toolkit were based on a review of randomized controlled trials rather than theory-based approach. Furthermore, the TAI toolkit was designed exclusively for patients with asthma and chronic obstructive pulmonary disease. Our study extends prior research by using behavioural theory and involving multiple stakeholders, including community pharmacists, physicians, and patients. The NMS toolkit could be adapted for different populations with different diseases in various settings.

Although there is a lack of theory-based interventions to promote medication adherence, the BCTs selected to address reasons for nonadherence were consistent with previous studies evaluating interventions related to promote medication adherence in acute coronary syndrome [49], in chronic disease [50] and type 2 diabetes [51]. The European Competence Framework to support behaviour change in persons self-managing chronic diseases suggested pros and cons as one of the selected BCTs to promote medication adherence as a part of Train4Health project [29]. In the present study, the community pharmacist found pros and cons inapplicable in daily practice because they thought this could be difficult in patients with low literacy. As noted in a previous study [52], this may be due to their thoughts about the impact and action of the pharmaceutical care interventions, which were considered barriers. In another previous study [53], health care providers’ lack of time and awareness about motivational interviewing were cited as barriers to implement motivational interviewing. Furthermore, the providers commented that they feared acute adverse effects of the medications when administered in the community pharmacy under their supervision. As a result, the proposed intervention related to exposure, which was one of the BCTs mapped on to reasons for nonadherence, was rated as inapplicable by the pharmacists.

This toolkit could be used to tailor pharmacist interventions following patient engagement in the NMS. In the protocolized follow-up call (within 7–10 days after starting a new medication or monthly), the three extent of nonadherence items on the DOSE Nonadherence scale could be used to explore whether or not the patient is adhering to the new medication. If not, the patient could be asked to rate the reasons for non-adherence in the toolkit to identify the ones that most contribute to nonadherence. The rating of each reason of nonadherence will empower and enable the patient in their care plan.

The next step in this research program is to conduct feasibility and evaluation studies (randomized controlled trials or quasi-experimental studies) to test the impact of the NMS toolkit on patient adherence and clinical outcomes. Prior to national implementation, community pharmacists who have identified themselves as early adopters may participate in these studies. Before starting the nationwide provision of NMS service in Türkiye, the continuing professional development program for community pharmacists should be designed with virtual and in-person options. The competence of pharmacists to support medication adherence could be improved by integrating this new toolkit and BCTs into pharmacy curricula [29]. In this way, they can generate personalized interventions to overcome nonadherence problems according to the reasons for nonadherence which are not available in this NMS toolkit. In addition to improving community pharmacists' knowledge of guideline-based disease management, this program should focus on improving community pharmacists' skills in patient education and counseling and in communicating with the patients to deliver these the pharmacist interventions in the NMS toolkit. In addition to primary care, this toolkit could be used in other settings. To improve the usability of this toolkit, a user-friendly version could be designed, including an application for mobile devices. Barriers related to implementing this service can be identified to generate implementation strategies to overcome them.

There are many strengths of this study. The UK Medical Research Council’s guidance was used to develop this NMS tool by considering the context, using a theory-based approach, and involving multiple stakeholders (patients, pharmacists, treating physicians) [54]. The use of multiple methods to assess content and construct validity is another strength of this study. However, there were some limitations of this study. First, the cognitive interviews and the Delphi study may have been subject to selection bias due to the sampling method used. Second, the responders of Delphi study were early adopters [55], who were expected to have high capacity and motivation to provide pharmaceutical care service to promote medication adherence. This might have resulted in high acceptance rate of proposed interventions in our study. Third, recall and social desirability biases may have occurred during cognitive interviews. Fourth, we may not have captured all relevant reasons for nonadherence in other populations, despite allowing participants the opportunity to add more that were not on the list. As the toolkit is tested in daily practice, the new reasons for non-adherence could be identified through the addition of an open-ended question to explore additional barriers, and pharmacist interventions could be adapted based on feedback from patients and community pharmacists. Fifth, although the pharmacists were trained to conduct the cognitive interviews, they could have introduced bias. Fifth, the results may not generalize to other samples. Due to practical constraints, we were not able to use a probability sampling method. In a previous study, lower education was associated with medication nonadherence, and there was no association by gender in previous study conducted in Turkish community pharmacies [56]. Additionally, female participants had a higher prevalence of hypertension and diabetes in the Turkish Health Research conducted by Turkish Statistical Institute [57].

## Conclusion

The NMS Toolkit was developed for use by community pharmacists to identify and address reasons for nonadherence in patients newly started on medications for hypertension, diabetes, or dyslipidemia. The toolkit includes screening for reasons for nonadherence and pharmacy interventions to address nonadherence issues. This toolkit will assist community pharmacists in developing personalized interventions to overcome nonadherence problems in patients newly starting medications. Further evaluation and implementation studies should be conducted to assess the impact of this new toolkit on patients' medication adherence levels and clinical outcomes.

## Supplementary Information

Below is the link to the electronic supplementary material.

Supplementary file1 (DOCX 55 kb)

## Data Availability

Data are available from the corresponding author on reasonable request.
