# Supplementary material for: Development of a behavioural theory-based New Medicine Service toolkit for community pharmacists to promote medication adherence
Source: Int J Clin Pharm. 2025 Jun 28;47(6):1794–811. doi: 10.1007/s11096-025-01959-3 (PMC12630297; doi:10.1007/s11096-025-01959-3)
Supplement: Supplementary file 1 — Supplementary file1 (DOCX 55 kb) [file 11096_2025_1959_MOESM1_ESM.docx]

**Development of a Behavioural Theory-Based New Medicine Service Toolkit for Community Pharmacists to Promote Medication Adherence**

Betul Okuyan^1,*^, Pınar Ay^2^, Mesut Sancar^1^, Vildan Özcan^3^, Ozge Durak-Albayrak^3^, Meltem Turker^3^, Arman Uney^3^, Corrine I Voils^4,5^

^1^Department of Clinical Pharmacy, Faculty of Pharmacy, Marmara University, Istanbul, Türkiye.

^2^Department of Public Health, Faculty of Medicine, Marmara University, Istanbul, Türkiye.

^3^Turkish Pharmacists' Association, Ankara, Türkiye.

^4^Department of Internal Medicine, School of Medicine, University of Utah, Salt Lake City, UT, USA.

^5^William S. Middleton Memorial Veterans Hospital, Madison, WI, USA.

*Corresponding author at Betul Okuyan. Marmara University Faculty of Pharmacy Clinical Pharmacy Department Istanbul, Türkiye e-mail address: betulokuyan@yahoo.com

**Supplement File 1.** Mapping reasons, Theoretical Domains Framework domains, and Behavioral Change Techniques for all items and consensus about applicability of proposed pharmacist interventions in New Medicine Service Toolkit

| **Items***  **I missed my dose because…** | **TDF Domain** | **BCTs** | **Proposed pharmacist interventions** | **Level of consensus**  **on applicability of proposed pharmacist intervention (n=25)** | | | | | |
| --- | --- | --- | --- | --- | --- | --- | --- | --- | --- |
|  |  |  |  | **First Round n (%)** | | | **Second Round n (%)** | | |
|  |  |  |  | **Agreement** | **Neutral** | **Disagreement** | **Agreement** | **Neutral** | **Disagreement** |
| 1. I did not know why I must take this medicine.^#^ | Knowledge | 5.1. Information about health consequences | NMS#1.1. Provide information regarding the health consequences of (taking/not taking) medications based on the Health Belief Model by using reliable references and written materials or a QR code with relevant material. | 20 (80) | 5 (20) | 0 (0) |  |  |  |
| 2. I did not know what the side effects or potential harm of this medicine were. ^#^ | Knowledge | 5.1. Information about health consequences | NMS#2.1. Provide information regarding potential side effects based on the Health Belief Model by using reliable references and written materials. *If there is no time for counselling, provide the patient with written materials or a QR code of relevant material.* | 17 (68) | 6 (24) | 2 (8) | 21 (84) | 4 (16) | 0 (0) |
| 3. I did not understand the physician’s/ pharmacist’s instructions. ^#^ | Knowledge | 4.1. Instruction on how to perform behaviour | NMS#3.1. Provide information by using the teach-back method using written materials and a short video. | 21 (84) | 1 (4) | 3 (12) |  |  |  |
|  |  | 8.1. Behavioural practice/rehearsal | NMS#3.2. Prompt the patient to practice using an insulin pen in the community pharmacy. | 19 (76) | 5 (20) | 1 (4) |  |  |  |
|  |  | 1.4 Action Planning | NMS#3.3. Discuss medication scheduling with the patient according to their daily routine by providing a pill card and/or pill box. | 22 (88) | 0 (0) | 3 (12) |  |  |  |
|  |  | 12.5. Adding objects to the environment | NMS#3.4. Provide a pill card and/or pill box. *NOTE: The patient's self-efficacy to use the pill box should be considered. Also, the pharmacist should assess the appropriateness of the pill box for the medications being used.* | 20 (80) | 4 (16) | 1 (4) |  |  |  |
| 4. I could not meet the food requirements (including empty stomach, taking with meals, no taking with alcohol). ^#^ | Skills | 1.4. Action Planning | NMS#4.1. Discuss medication scheduling with the patient according to their daily routine (if the patient has no plan). | 20 (80) | 2 (8) | 3 (12) |  |  |  |
|  |  | 12.5. Adding objects to the environment | NMS#4.2. Provide a medication schedule to the patient (including a pill card) and suggest using a pill box (if the patient has no plan). *NOTE: The patient's self-efficacy to use the pill box should be considered. Also, the pharmacist should assess the appropriateness of the pill box for the medications being used.* | 17 (68) | 4 (16) | 4 (16) | 21 (84) | 2 (8) | 2 (8) |
|  |  | 8.3. Habit formation (If the patient has schedule/plan) | NMS#4.3. Advise the patient to integrate the behaviour with existing habits, if possible (if the patient has plan). | 20 (80) | 2 (8) | 3 (12) |  |  |  |
|  |  | 4.1. Instruction on how to perform behaviour | NMS#4.4. Provide patient education and counselling by using the teach-back method. Discuss alcohol intake (including the amounts). | 14 (56) | 8 (32) | 3 (12) | 22 (88) | 2 (8) | 1 (4) |
|  |  | 7.1. Prompts/cues (If the patient has schedule/plan) | NMS#4.5. Advise the patient to set an alarm and place a sticker or magnet to remind them to take their medication. | 22 (88) | 2 (8) | 1 (4) |  |  |  |
|  |  | 12.1. Restructuring the physical environmental (If the patient has schedule/plan) | NMS#4.6. Advise the patient about where to put the pill box to promote medication taking, such as on the kitchen table or next to the bed. | 20 (80) | 5 (20) | 0 (0) |  |  |  |
| 5. My treatment was so complicated, because I was on so many medications with different instructions. ^#^ | Beliefs about capabilities | 1.2. Problem solving | NMS#5.1. Provide a Brown Bag check-up by using the teach-back method.  Contact the physician to simplify the regimen. | 20 (80) | 1 (4) | 4 (16) | 21 (84) | 3 (12) | 1 (4) |
|  |  | 1.4. Action Planning | NMS#5.2. If required, provide a medication schedule to the patient (including a pill card) and suggest using a pill box. | 21 (84) | 2 (8) | 2 (8) |  |  |  |
|  |  | 12.5. Adding objects to the environment | NMS#5.3. Provide a pill card and/or pill box. *NOTE: The patient's self-efficacy to use the pill box should be considered. Also, the pharmacist should assess the appropriateness of the pill box for the medications being used.* | 20 (80) | 4 (16) | 1 (4) |  |  |  |
| 6. The medication caused side effects. ^#^ | Reinforcement | 1.2. Problem solving | NMS#6.1. Contact the patient’s physician to modify the treatment or refer them to their physician. | 20 (80) | 4 (16) | 1 (4) |  |  |  |
|  |  | 5.1. Information about health consequences | NMS#6.2. Provide information regarding management of potential side effects by using reliable references *giving written materials or a QR code for relevant material.* | 17 (68) | 6 (24) | 2 (8) | 20 (80) | 3 (12) | 2 (8) |
| 7. I did not remember to take my medicine. ^#^ | Memory, attention, and decision processes | 2.3. Self-monitoring of behaviour | NMS#7.1. Advise the patient to use a diary or checklist. | 21 (84) | 3 (12) | 1 (4) |  |  |  |
|  |  | 8.3. Habit formation | NMS#7.2. Advise the patient to integrate the behaviour with existing habits, if possible. | 21 (84) | 1 (4) | 3 (12) |  |  |  |
|  |  | 12.1. Restructuring the physical environmental | NMS#7.3. Advise the patient about where to put the pill box to promote medication taking, such as on the kitchen table or next to the bed. | 22 (88) | 2 (8) | 1 (4) |  |  |  |
|  |  | 7.1. Prompts/cues | NMS#7.4. Advise the patient to set an alarm and place a sticker or magnet to remind them to take their medication. | 23 (92) | 0 (0) | 2 (8) |  |  |  |
|  |  | 12.5. Adding objects to the environment | NMS#7.5. Advise the patient to use a pill box. *NOTE: The patient's self-efficacy to use the pill box should be considered. Also, the pharmacist should assess the appropriateness of the pill box for the medications being used.* | 21 (84) | 3 (12) | 1 (4) |  |  |  |
|  |  | 3.2. Social support (practical) | NMS#7.6. Advise asking a friend/family member to help the patient remember to take their medication. *NOTE: If there is no time for counselling, provide the patient with written materials or a QR code with relevant material.* | 17 (68) | 6 (24) | 2 (8) | 21 (84) | 3 (12) | 1 (4) |
| 8. I could not fill the prescription on time. ^#^ | Environmental context and resources | 3.2. Social support (practical) | NMS#8.1. Advise asking a friend/family member to help the patient take their medication. *NOTE: If there is no time for counselling, provide the patient with written materials or a QR code of relevant material.* | 18 (72) | 3 (12) | 4 (16) | 19 (76) | 3 (12) | 3 (12) |
|  |  | 7.1. Prompts/cues | NMS#8.2. Advise the patient to set an alarm and place a sticker or magnet to remind them to take their medication. | 16 (64) | 3 (12) | 6 (24) | 18 (72) | 4 (16) | 3 (12) |
| 9. I could not get my medicine at pharmacy. ^#^ | Environmental context and resources | 1.2. Problem solving | NMS#9.1. Identify the reasons (such as medication shortage). Advise the patient to use generic medications and/or contact their physician. | 22 (88) | 1 (4) | 2 (8) |  |  |  |
| 10. I could not remember whether I took my medicine or not. ^#^ | Memory, attention, and decision processes | 2.3. Self-monitoring of behaviour | NMS#10.1. Advise the patient to use a diary or checklist. | 21 (84) | 2 (8) | 2 (8) |  |  |  |
|  |  | 12.5. Adding objects to the environment | NMS#10.2. Advise the patient to use a pill box. *NOTE: The patient's self-efficacy to use the pill box should be considered. Also, the pharmacist should assess the appropriateness of the pill box for the medications being used.* | 19 (76) | 5 (20) | 1 (4) |  |  |  |
| 11. The physician did not spend enough time with me and did not explain my treatment. ^#^ | Social influences | 5.1. Information about health consequences | NMS#11.1. Provide information regarding the health consequences of (taking/not taking) medications based on the Health Belief Model by using reliable references and written materials. | 18 (72) | 5 (20) | 2 (8) |  |  |  |
|  |  | 8.1. Behavioural practice/rehearsal | EXCLUDED: Discuss how to best prepare for and behave during healthcare visits. Ask the patient if they have questions and answer the questions. List the questions the patient could ask at their next physician appointment. | 19 (76) | 2 (8) | 4 (16) | 17 (68) | 6 (24) | 2 (8) |
|  |  | 9.1. Credible source | EXCLUDED: Provide information regarding management of the condition using national and international guidelines. This could be a short video. *NOTE* If there is no time for counselling, provide the patient with written materials or a QR code with relevant material. | 8 (32) | 4 (16) | 13 (52) | 10 (40) | 5 (20) | 10 (40) |
| 12. I could not get answers to my questions about the medication. ^#^ | Social influences | 1.2. Problem solving | NMS#12.1. Ask the patient if they have questions and answer the questions. If required, refer them to their physician. | 21 (84) | 3 (12) | 1 (4) |  |  |  |
|  |  | 8.1. Behavioural practice/rehearsal | EXCLUDED: Discuss how to best prepare for and behave during healthcare visits. Ask the patient if they have questions and answer the questions. List the questions the patient could ask at their next physician appointment. | 17 (68) | 5 (20) | 3 (12) | 19 (76) | 2 (8) | 4 (16) |
| 13. I went to another physician, and this physician said I didn't need to use this medicine. ^#^ | Social influences | 2.4. Monitoring of outcome(s) of behaviour without feedback | NMS#13.1. Advise the patient to record their blood pressure/glucose daily or to have their lipid profile checked one month later. | 22 (88) | 3 (12) | 0 (0) |  |  |  |
| 14. I am worried about side effects.^#^ | Emotion  Belief of consequences | 5.1. Information about health consequences | NMS#14.1. Provide the patient with counselling regarding potential side effects and their management using reliable references. | 19 (76) | 5 (20) | 1 (4) |  |  |  |
|  |  | 9.2. Pros and cons | EXCLUDED: Use motivational interviewing to have patients generate a list of pros and cons of using medications (if required, the pharmacist can facilitate the process). *NOTE: This intervention may be recommended for patients with an intentional (deliberate) non-adherence problem and/or patients with high health literacy.* | 8 (32) | 7 (28) | 10 (40) | 8 (32) | 9 (36) | 8 (32) |
| 15. I did not receive any practical training on how to use special medications (such as insulin pens). | Skills | 4.1. Instruction on how to perform behaviour | NMS#15.1. Provide information by using the teach-back method using written materials and a short video. | 20 (80) | 2 (8) | 3 (12) |  |  |  |
|  |  | 8.1. Behavioural practice/rehearsal | NMS#15.2. Prompt the patients to practice using an insulin pen in the community pharmacy. | 21 (84) | 3 (12) | 1 (4) |  |  |  |
| 16. I do not like needles. | Emotion  Belief of consequences | 8.1. Behavioural practice/rehearsal | NMS#16.1. Check whether the patient took the medication correctly. | 21 (84) | 1 (4) | 3 (12) |  |  |  |
|  |  | 1.2. Problem solving | NMS#16.2. Contact/refer the patient to the physician to assess the medication treatment plan. (The physician would assess the use of oral medications instead of SC medication.) | 20 (80) | 2 (8) | 3 (12) |  |  |  |
|  |  | 3.2. Social support (practical) | NMS#16.3. Advise asking a friend/family member to help the patient administer SC medications. *NOTE: If there is no time for counselling, provide the patient with written materials or a QR code with relevant material.* | 15 (60) | 7 (28) | 3 (12) | 20 (80) | 4 (16) | 1 (4) |
|  |  | 9.2. Pros and cons | EXCLUDED: Use motivational interviewing to have patients generate a list of pros and cons of using medications (if required, the pharmacist can facilitate the process). *NOTE: This intervention may be recommended for patients with an intentional (deliberate) non-adherence problem and/or patients with high health literacy.* | 12 (48) | 5 (20) | 8 (32) | 9 (36) | 14 (56) | 2 (8) |
|  |  | 7.7. Exposure | EXCLUDED: Agree on administration of the SC medications by the patient *under supervision of the health care provider* until the patient feels confident. | 14 (56) | 4 (16) | 7 (28) | 15 (60) | 9 (36) | 1 (4) |
| 17. I hate to take the medicine. | Emotion  Belief of consequences | 3.2. Social support (practical) | NMS#17.1. Advise asking a friend/family member to help the patient with subcutaneous administration of the medication. *NOTE: If there is no time for counselling, provide the patient with written materials or a QR code with relevant material.* | 15 (60) | 6 (24) | 4 (16) | 19 (76) | 5 (20) | 1 (4) |
|  |  | 9.2. Pros and cons | EXCLUDED: Use motivational interviewing to have patients generate a list of pros and cons of using medications (if required, the pharmacist can facilitate the process). *NOTE: This intervention may be recommended for patients with an intentional (deliberate) non-adherence problem and/or patients with high health literacy.* | 12 (48) | 7 (28) | 6 (24) | 14 (56) | 8 (32) | 3 (12) |
|  |  | 7.7. Exposure | EXCLUDED: Agree on administration of the SC medications by the patient *under supervision of health care provider* until the patient feels confident. | 13 (52) | 4 (16) | 8 (32) | 15 (60) | 6 (24) | 4 (16) |
| 18. I was afraid the medication would interact with other medication I take. | Belief of consequences | 5.1. Information about health consequences | NMS#18.1. Provide information regarding drug interactions and their management using reliable references. | 22 (88) | 1 (4) | 2 (8) |  |  |  |
|  |  | 9.2. Pros and cons | EXCLUDED: Use motivational interviewing to have patients generate a list of pros and cons of using medications (if required, the pharmacist can facilitate the process). *NOTE: This intervention may be recommended for patients with an intentional (deliberate) non-adherence problem and/or patients with high health literacy.* | 12 (48) | 9 (36) | 4 (16) | 13 (52) | 7 (28) | 5 (20) |
| 19. I thought treatment was over. | Belief of consequences | 1.5. Review behaviour goal(s) | NMS#19.1. Discuss treatment goals using national and international guidelines. | 19 (76) | 4 (16) | 2 (8) |  |  |  |
|  |  | 5.1. Information about health consequences | NMS#19.2. Discuss goals of medication taking. *NOTE: If there is no time for counselling, provide the patient with written materials or a QR code with relevant material.* | 15 (60) | 6 (24) | 4 (16) | 22 (88) | 2 (8) | 1 (4) |
| 20. The medication was not working. | Belief of consequences | 2.6 Biofeedback  2.3. Self-monitoring of behaviour  2.7. Feedback on outcome(s) of behaviour | NMS#20.1. Advise the patient to self-monitor clinical outcomes by using a glucometer and/or blood pressure monitor and recording the values.  Advise the patient to record daily medication taking using a diary.  Schedule a meeting to discuss the patient’s blood pressure and glucose values according to their weekly record.  Inform the patient about the decrease in their blood pressure/glucose when they take the medication and the increase when they do not.  *NOTE: This intervention may be recommended for patients with an intentional (deliberate) non-adherence problem and/or patients with high health literacy.* | 20 (80) | 2 (8) | 3 (12) |  |  |  |
|  |  | 5.1. Information about health consequences | NMS#20.2. Provide information about impact of the medications using reliable references. | 20 (80) | 3 (12) | 2 (8) |  |  |  |
| 21. I did not think I needed the medicine | Belief of consequences | 2.6 Biofeedback  2.3. Self-monitoring of behaviour  2.7. Feedback on outcome(s) of behaviour | NMS#21.1. Advise the patient to self-monitor clinical outcomes by using a glucometer and/or blood pressure monitor and recording the measures. Advise the patient to record daily medication taking using a diary. Schedule a meeting to discuss the patient’s blood pressure and glucose values according to their weekly record.  Inform the patient about the decrease in their blood pressure/glucose when they take the medication and the increase when they do not.  *NOTE: This intervention may be recommended for patients with an intentional (deliberate) non-adherence problem and/or patients with high health literacy.* | 15 (60) | 7 (28) | 3 (12) | 21 (84) | 2 (8) | 2 (8) |
|  |  | 5.1. Information about health consequences | NMS#21.2. Provide information regarding the health consequences of (taking/not taking) medications based on the Health Belief Model. | 20 (80) | 3 (12) | 2 (8) |  |  |  |
|  |  | 9.2. Pros and cons | EXCLUDED: Use motivational interviewing to have patients generate a list of pros and cons of using medications (if required, the pharmacist can facilitate the process). *NOTE: This intervention may be recommended for patients with an intentional (deliberate) non-adherence problem and/or patients with high health literacy.* | 14 (56) | 6 (24) | 5 (20) | 13 (52) | 10 (40) | 2 (8) |
| 22. The injection causes pain. | Reinforcement | 5.1. Information about health consequences | NMS#22.1. Provide information about why the injection causes pain and potential side effects. | 23 (92) | 1 (4) | 1 (4) |  |  |  |
|  |  | 8.1. Behavioural practice/rehearsal | NMS#22.2. Check whether the patient applied correctly using the teach-back method. | 22 (88) | 2 (8) | 1 (4) |  |  |  |
|  |  | 1.2. Problem solving | NMS#22.3. Contact/refer the patient to the physician to assess the medication treatment plan. (The physician would assess the use of oral medications instead of SC medication) | 16 (64) | 6 (24) | 3 (12) | 21 (84) | 2 (8) | 2 (8) |
|  |  | 4.1. Instruction on how to perform behaviour | EXCLUDED: Provide information on how to administer injections accurately. | 14 (56) | 7 (28) | 4 (16) | 16 (64) | 6 (24) | 3 (12) |
| 23. The medicine was making my condition worse. | Reinforcement | 5.1. Information about health consequences | NMS#23.1. Provide information regarding the health consequences of (taking/not taking) medications based on the Health Belief Model and using reliable sources. | 18 (72) | 5 (20) | 2 (8) |  |  |  |
|  |  | 2.6 Biofeedback | NMS#23.2. Advise the patient to self-monitor clinical outcomes by using a glucometer and/or blood pressure monitor and recording the values. | 18 (72) | 4 (16) | 3 (12) |  |  |  |
|  |  | 2.3. Self-monitoring of behaviour | NMS#23.3. Advise the patient to record daily medication taking using a diary. | 18 (72) | 3 (12) | 4 (16) | 19 (76) | 4 (16) | 2 (8) |
|  |  | 2.7. Feedback on outcome(s) of behaviour | NMS#23.4. Schedule a meeting to discuss the patient’s blood pressure and glucose values according to their weekly record. Inform the patient the decrease in their blood pressure/glucose when they take the medication and the increase when they do not. | 18 (72) | 5 (20) | 2 (8) |  |  |  |
| 24. I was busy. | Goals | 1.4. Action Planning | NMS#24.1. Discuss medication scheduling with the patient according to their daily routine. | 20 (80) | 3 (12) | 2 (8) |  |  |  |
|  |  | 12.5. Adding objects to the environment | NMS#24.2. Provide a pill card and/or pill box. *NOTE: The patient's self-efficacy to use the pill box should be considered. Also, the pharmacist should assess the appropriateness of the pill box for the medications being used.* | 16 (64) | 7 (28) | 2 (8) | 23 (92) | 2 (8) | 0 (0) |
|  |  | 8.3. Habit formation | NMS#24.3. Advise the patient to integrate the behaviour with existing habits, if possible. | 18 (72) | 4 (16) | 3 (12) |  |  |  |
|  |  | 12.1. Restructuring the physical environmental | NMS#24.4. Advise the patient about where to put the pill box to promote medication taking, such as on the kitchen table or next to the bed. | 18 (72) | 6 (24) | 1 (4) |  |  |  |
|  |  | 7.1. Prompts/cues | NMS#25.5. Advise the patient to set an alarm and place a sticker or magnet to remind them to take their medication. | 19 (76) | 4 (16) | 2 (8) |  |  |  |
|  |  | 3.2. Social support (practical) | NMS#25.6. Advise asking a friend/family member to help the patient remember to take their medication. *NOTE: If there is no time for counselling, provide the patient with written materials or a QR code with relevant material.* | 17 (68) | 4 (16) | 4 (16) | 21 (84) | 4 (16) | 0 (0) |
| 25. The medicine was too expensive. | Environmental context and resources | 1.2. Problem solving | NMS#25.1. Identify the reasons and solutions. Advise the patient to use generic medications and/or contact their physician. | 17 (68) | 6 (24) | 2 (8) | 21 (84) | 4 (16) | 0 (0) |
| 26. I did not have my medicines with me. | Environmental context and resources | 3.2. Social support (practical) | NMS#26.1. Advise asking a friend/family member to help the patient take their medication. *NOTE: If there is no time for counselling, provide the patient with written materials or a QR code with relevant material.* | 15 (60) | 7 (28) | 3 (12) | 18 (72) | 7 (28) | 0 (0) |
|  |  | 7.1. Prompts/cues | NMS#26.2. Advise the patient to set an alarm and place a sticker or magnet to remind them to take their medication. | 17 (68) | 4 (16) | 4 (16) | 18 (72) | 6 (24) | 1 (4) |
|  |  | 12.1. Restructuring the physical environment | NMS#26.3. Advise the patient to put the medications in the a bag/pill box or to keep spare medications. | 21 (84) | 3 (12) | 1 (4) |  |  |  |
| 27. Suitable conditions for storing my medicine were not available (such as refrigerator, humidity). | Environmental context and resources | 12.1. Restructuring the physical environment | NMS#27.1. Advise the patient to put the medications in the a bag/pill box or to keep spare medications. | 21 (84) | 2 (8) | 2 (8) |  |  |  |
| 28. I did not have the supplies needed to take the medicine (e.g., syringes/needles). | Environmental context and resources | 7.1. Prompts/cues | NMS#28.1. Advise the patient to set an alarm and place a sticker or magnet to remind them to take their medication. | 14 (56) | 6 (24) | 5 (20) | 18 (72) | 7 (28) | 0 (0) |
|  |  | 12.1. Restructuring the physical environment | NMS#28.2. Advise the patient to put the medications in a bag/pill box or to keep spare supplies. | 22 (88) | 1 (4) | 2 (8) |  |  |  |
|  |  | 3.2. Social support (practical) | EXCLUDED: Advise family/friends supporting the patient *by giving them written materials or a QR code with relevant material* | 11 (44) | 10 (40) | 4 (16) | 16 (64) | 8 (32) | 1 (4) |
| 29. There was no one who could help me take and use medications. | Social influences | 3.2. Social support (practical) | NMS#29.1. Provide pharmacist-led practical help.  Advise family/friends supporting the patient *by giving them written materials or a QR code with relevant material.* | 15 (60) | 4 (16) | 6 (24) | 18 (72) | 4 (16) | 3 (12) |
| 30. My family or friends suggested me not take the medicine. | Social influences | 5.1. Information about health consequences | NMS#30.1. Provide information regarding the health consequences of (taking/not taking) medications based on the Health Belief Model by using reliable references and *written materials or a QR code with relevant material.* | 18 (72) | 3 (12) | 4 (16) | 19 (76) | 6 (24) | 0 (0) |
|  |  | 9.1. Credible source | EXCLUDED: Provide information regarding management of the condition using national and international guidelines. This could be a short video. *NOTE:* If there is no time for counselling, provide the patient with written materials or a QR code with relevant material. | 11 (44) | 7 (28) | 7 (28) | 14 (56) | 5 (20) | 6 (24) |
| 31. I hesitated to ask my questions to the physician about the medicines. | Social influences | 8.1. Behavioural practice/rehearsal | NMS#31.1. Discuss how to best prepare and behave during healthcare visits. Ask the patient if they have questions and answer the questions. List the questions the patient could ask and their next physician appointment. | 18 (72) | 4 (16) | 3 (12) |  |  |  |

* All items are translated from Turkish. **Community pharmacists rated the applicability of the proposed pharmacist interventions by considering practical aspects of the pharmacy workflow, workload, and patient needs. ^#^Items included in short form. In the modified Delphi study, the changes made after round 1 are presented in italics. Theoretical Domains Framework (TDF); Behavioral Change Techniques (BCT); New Medicine Service (NMS).
